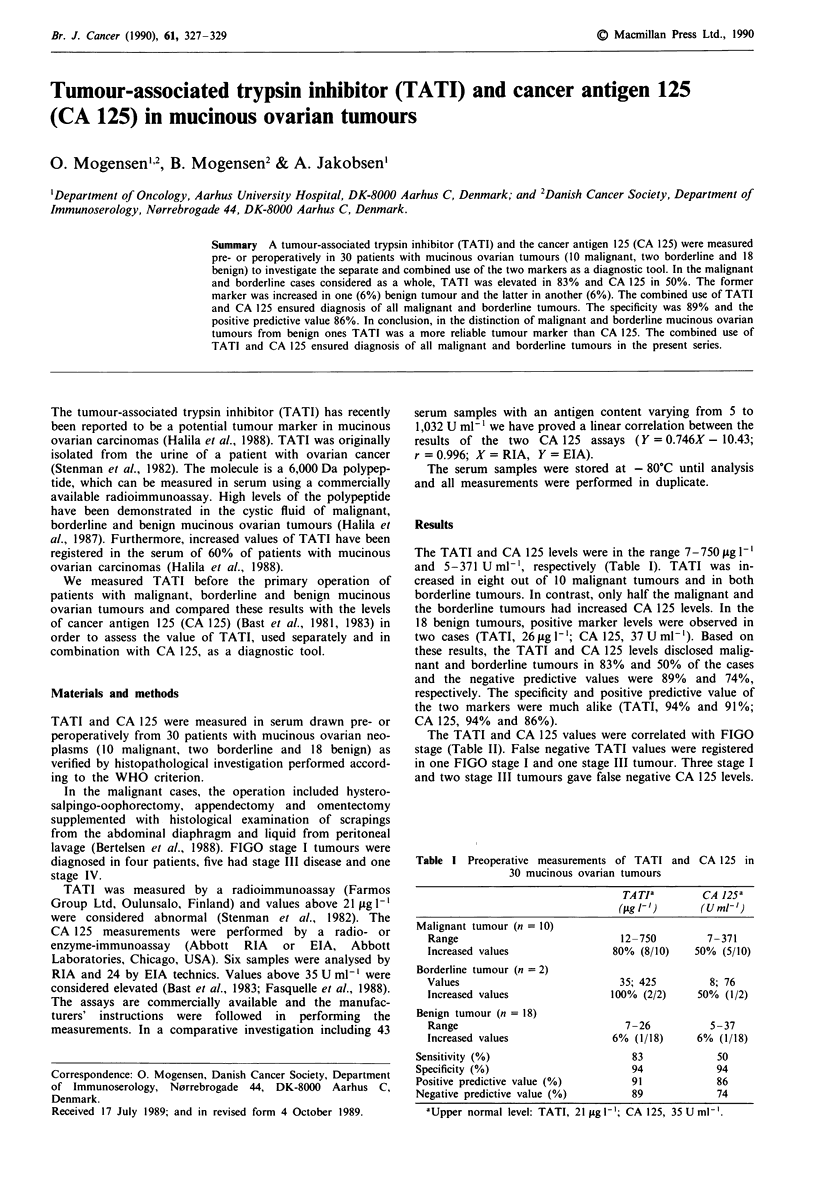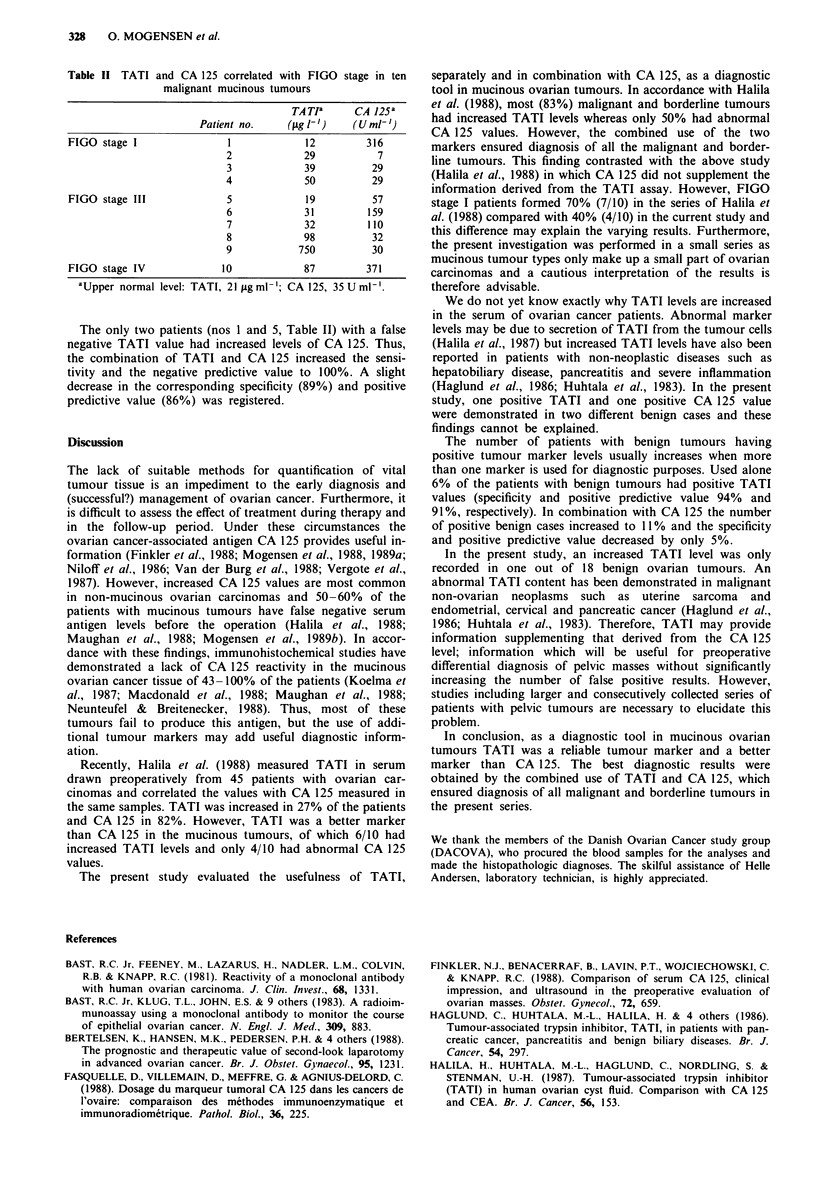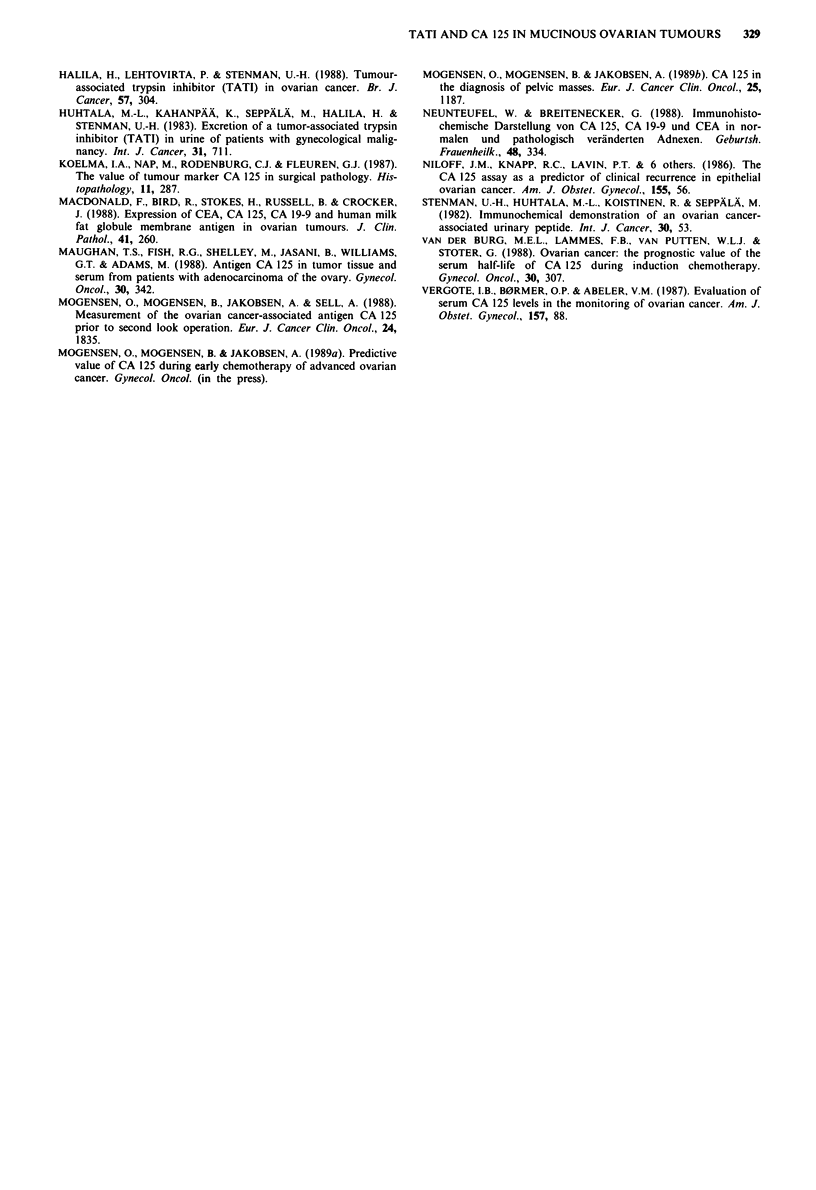# Tumour-associated trypsin inhibitor (TATI) and cancer antigen 125 (CA 125) in mucinous ovarian tumours.

**DOI:** 10.1038/bjc.1990.64

**Published:** 1990-02

**Authors:** O. Mogensen, B. Mogensen, A. Jakobsen

**Affiliations:** Department of Oncology, Aarhus University Hospital, Denmark.

## Abstract

A tumour-associated trypsin inhibitor (TATI) and the cancer antigen 125 (CA 125) were measured pre- or peroperatively in 30 patients with mucinous ovarian tumours (10 malignant, two borderline and 18 benign) to investigate the separate and combined use of the two markers as a diagnostic tool. In the malignant and borderline cases considered as a whole, TATI was elevated in 83% and CA 125 in 50%. The former marker was increased in one (6%) benign tumour and the latter in another (6%). The combined use of TATI and CA 125 ensured diagnosis of all malignant and borderline tumours. The specificity was 89% and the positive predictive value 86%. In conclusion, in the distinction of malignant and borderline mucinous ovarian tumours from benign ones TATI was a more reliable tumour marker than CA 125. The combined use of TATI and CA 125 ensured diagnosis of all malignant and borderline tumours in the present series.


Br. J. Cancer (1990), 61, 327-329

© Macmillan Press Ltd., 1990

### Tumour-associated trypsin inhibitor (TATI) and cancer antigen 125 (CA 125) in mucinous ovarian tumours

O. Mogensen[1,2], B. Mogensen[2] & A. Jakobsen[1]

[1]*Department of Oncology, Aarhus University Hospital, DK-8000 Aarhus C, Denmark; and* [2]*Danish Cancer Society, Department of Immunoserology, Nørrebrogade 44, DK-8000 Aarhus C, Denmark.*

**Summary** A tumour-associated trypsin inhibitor (TATI) and the cancer antigen 125 (CA 125) were measured pre- or peroperatively in 30 patients with mucinous ovarian tumours (10 malignant, two borderline and 18 benign) to investigate the separate and combined use of the two markers as a diagnostic tool. In the malignant and borderline cases considered as a whole, TATI was elevated in 83% and CA 125 in 50%. The former marker was increased in one (6%) benign tumour and the latter in another (6%). The combined use of TATI and CA 125 ensured diagnosis of all malignant and borderline tumours. The specificity was 89% and the positive predictive value 86%. In conclusion, in the distinction of malignant and borderline mucinous ovarian tumours from benign ones TATI was a more reliable tumour marker than CA 125. The combined use of TATI and CA 125 ensured diagnosis of all malignant and borderline tumours in the present series.

The tumour-associated trypsin inhibitor (TATI) has recently been reported to be a potential tumour marker in mucinous ovarian carcinomas (Halila *et al.*, 1988). TATI was originally isolated from the urine of a patient with ovarian cancer (Stenman *et al.*, 1982). The molecule is a 6,000 Da polypeptide, which can be measured in serum using a commercially available radioimmunoassay. High levels of the polypeptide have been demonstrated in the cystic fluid of malignant, borderline and benign mucinous ovarian tumours (Halila *et al.*, 1987). Furthermore, increased values of TATI have been registered in the serum of 60% of patients with mucinous ovarian carcinomas (Halila *et al.*, 1988).

We measured TATI before the primary operation of patients with malignant, borderline and benign mucinous ovarian tumours and compared these results with the levels of cancer antigen 125 (CA 125) (Bast *et al.*, 1981, 1983) in order to assess the value of TATI, used separately and in combination with CA 125, as a diagnostic tool.

### Materials and methods

TATI and CA 125 were measured in serum drawn pre- or peroperatively from 30 patients with mucinous ovarian neoplasms (10 malignant, two borderline and 18 benign) as verified by histopathological investigation performed according to the WHO criterion.

In the malignant cases, the operation included hystero-salpingo-oophorectomy, appendectomy and omentectomy supplemented with histological examination of scrapings from the abdominal diaphragm and liquid from peritoneal lavage (Bertelsen *et al.*, 1988). FIGO stage I tumours were diagnosed in four patients, five had stage III disease and one stage IV.

TATI was measured by a radioimmunoassay (Farmos Group Ltd, Oulunsalo, Finland) and values above 21 $\mu g\,l^{-1}$ were considered abnormal (Stenman *et al.*, 1982). The CA 125 measurements were performed by a radio- or enzyme-immunoassay (Abbott RIA or EIA, Abbott Laboratories, Chicago, USA). Six samples were analysed by RIA and 24 by EIA technics. Values above 35 $U\,ml^{-1}$ were considered elevated (Bast *et al.*, 1983; Fasquelle *et al.*, 1988). The assays are commercially available and the manufacturers' instructions were followed in performing the measurements. In a comparative investigation including 43 serum samples with an antigen content varying from 5 to 1,032 $U\,ml^{-1}$ we have proved a linear correlation between the results of the two CA 125 assays ($Y = 0.746X - 10.43$; $r = 0.996$; $X$ = RIA, $Y$ = EIA).

The serum samples were stored at − 80°C until analysis and all measurements were performed in duplicate.

### Results

The TATI and CA 125 levels were in the range 7–750 $\mu g\,l^{-1}$ and 5–371 $U\,ml^{-1}$, respectively (Table I). TATI was increased in eight out of 10 malignant tumours and in both borderline tumours. In contrast, only half the malignant and the borderline tumours had increased CA 125 levels. In the 18 benign tumours, positive marker levels were observed in two cases (TATI, 26 $\mu g\,l^{-1}$; CA 125, 37 $U\,ml^{-1}$). Based on these results, the TATI and CA 125 levels disclosed malignant and borderline tumours in 83% and 50% of the cases and the negative predictive values were 89% and 74%, respectively. The specificity and positive predictive value of the two markers were much alike (TATI, 94% and 91%; CA 125, 94% and 86%).

The TATI and CA 125 values were correlated with FIGO stage (Table II). False negative TATI values were registered in one FIGO stage I and one stage III tumour. Three stage I and two stage III tumours gave false negative CA 125 levels.

**Table I** Preoperative measurements of TATI and CA 125 in 30 mucinous ovarian tumours

| | *TATI*[a] ($\mu g\,l^{-1}$) | *CA 125*[a] ($U\,ml^{-1}$) |
|---|---|---|
| Malignant tumour ($n$ = 10) | | |
| Range | 12–750 | 7–371 |
| Increased values | 80% (8/10) | 50% (5/10) |
| Borderline tumour ($n$ = 2) | | |
| Values | 35; 425 | 8; 76 |
| Increased values | 100% (2/2) | 50% (1/2) |
| Benign tumour ($n$ = 18) | | |
| Range | 7–26 | 5–37 |
| Increased values | 6% (1/18) | 6% (1/18) |
| Sensitivity (%) | 83 | 50 |
| Specificity (%) | 94 | 94 |
| Positive predictive value (%) | 91 | 86 |
| Negative predictive value (%) | 89 | 74 |

[a]Upper normal level: TATI, 21 $\mu g\,l^{-1}$; CA 125, 35 $U\,ml^{-1}$.

Correspondence: O. Mogensen, Danish Cancer Society, Department of Immunoserology, Nørrebrogade 44, DK-8000 Aarhus C, Denmark.

Received 17 July 1989; and in revised form 4 October 1989.

**Table II** TATI and CA 125 correlated with FIGO stage in ten malignant mucinous tumours

| | Patient no. | TATI[a] ($\mu g\ l^{-1}$) | CA 125[a] ($U\ ml^{-1}$) |
|---|---|---|---|
| FIGO stage I | 1 | 12 | 316 |
| | 2 | 29 | 7 |
| | 3 | 39 | 29 |
| | 4 | 50 | 29 |
| FIGO stage III | 5 | 19 | 57 |
| | 6 | 31 | 159 |
| | 7 | 32 | 110 |
| | 8 | 98 | 32 |
| | 9 | 750 | 30 |
| FIGO stage IV | 10 | 87 | 371 |

[a]Upper normal level: TATI, 21 $\mu g\ ml^{-1}$; CA 125, 35 $U\ ml^{-1}$.

The only two patients (nos 1 and 5, Table II) with a false negative TATI value had increased levels of CA 125. Thus, the combination of TATI and CA 125 increased the sensitivity and the negative predictive value to 100%. A slight decrease in the corresponding specificity (89%) and positive predictive value (86%) was registered.

### Discussion

The lack of suitable methods for quantification of vital tumour tissue is an impediment to the early diagnosis and (successful?) management of ovarian cancer. Furthermore, it is difficult to assess the effect of treatment during therapy and in the follow-up period. Under these circumstances the ovarian cancer-associated antigen CA 125 provides useful information (Finkler *et al.*, 1988; Mogensen *et al.*, 1988, 1989*a*; Niloff *et al.*, 1986; Van der Burg *et al.*, 1988; Vergote *et al.*, 1987). However, increased CA 125 values are most common in non-mucinous ovarian carcinomas and 50–60% of the patients with mucinous tumours have false negative serum antigen levels before the operation (Halila *et al.*, 1988; Maughan *et al.*, 1988; Mogensen *et al.*, 1989*b*). In accordance with these findings, immunohistochemical studies have demonstrated a lack of CA 125 reactivity in the mucinous ovarian cancer tissue of 43–100% of the patients (Koelma *et al.*, 1987; Macdonald *et al.*, 1988; Maughan *et al.*, 1988; Neunteufel & Breitenecker, 1988). Thus, most of these tumours fail to produce this antigen, but the use of additional tumour markers may add useful diagnostic information.

Recently, Halila *et al.* (1988) measured TATI in serum drawn preoperatively from 45 patients with ovarian carcinomas and correlated the values with CA 125 measured in the same samples. TATI was increased in 27% of the patients and CA 125 in 82%. However, TATI was a better marker than CA 125 in the mucinous tumours, of which 6/10 had increased TATI levels and only 4/10 had abnormal CA 125 values.

The present study evaluated the usefulness of TATI, separately and in combination with CA 125, as a diagnostic tool in mucinous ovarian tumours. In accordance with Halila *et al.* (1988), most (83%) malignant and borderline tumours had increased TATI levels whereas only 50% had abnormal CA 125 values. However, the combined use of the two markers ensured diagnosis of all the malignant and borderline tumours. This finding contrasted with the above study (Halila *et al.*, 1988) in which CA 125 did not supplement the information derived from the TATI assay. However, FIGO stage I patients formed 70% (7/10) in the series of Halila *et al.* (1988) compared with 40% (4/10) in the current study and this difference may explain the varying results. Furthermore, the present investigation was performed in a small series as mucinous tumour types only make up a small part of ovarian carcinomas and a cautious interpretation of the results is therefore advisable.

We do not yet know exactly why TATI levels are increased in the serum of ovarian cancer patients. Abnormal marker levels may be due to secretion of TATI from the tumour cells (Halila *et al.*, 1987) but increased TATI levels have also been reported in patients with non-neoplastic diseases such as hepatobiliary disease, pancreatitis and severe inflammation (Haglund *et al.*, 1986; Huhtala *et al.*, 1983). In the present study, one positive TATI and one positive CA 125 value were demonstrated in two different benign cases and these findings cannot be explained.

The number of patients with benign tumours having positive tumour marker levels usually increases when more than one marker is used for diagnostic purposes. Used alone 6% of the patients with benign tumours had positive TATI values (specificity and positive predictive value 94% and 91%, respectively). In combination with CA 125 the number of positive benign cases increased to 11% and the specificity and positive predictive value decreased by only 5%.

In the present study, an increased TATI level was only recorded in one out of 18 benign ovarian tumours. An abnormal TATI content has been demonstrated in malignant non-ovarian neoplasms such as uterine sarcoma and endometrial, cervical and pancreatic cancer (Haglund *et al.*, 1986; Huhtala *et al.*, 1983). Therefore, TATI may provide information supplementing that derived from the CA 125 level; information which will be useful for preoperative differential diagnosis of pelvic masses without significantly increasing the number of false positive results. However, studies including larger and consecutively collected series of patients with pelvic tumours are necessary to elucidate this problem.

In conclusion, as a diagnostic tool in mucinous ovarian tumours TATI was a reliable tumour marker and a better marker than CA 125. The best diagnostic results were obtained by the combined use of TATI and CA 125, which ensured diagnosis of all malignant and borderline tumours in the present series.

We thank the members of the Danish Ovarian Cancer study group (DACOVA), who procured the blood samples for the analyses and made the histopathologic diagnoses. The skilful assistance of Helle Andersen, laboratory technician, is highly appreciated.

### References

BAST, R.C. Jr, FEENEY, M., LAZARUS, H., NADLER, L.M., COLVIN, R.B. & KNAPP, R.C. (1981). Reactivity of a monoclonal antibody with human ovarian carcinoma. *J. Clin. Invest.*, **68**, 1331.

BAST, R.C. Jr, KLUG, T.L., JOHN, E.S. & 9 others (1983). A radioimmunoassay using a monoclonal antibody to monitor the course of epithelial ovarian cancer. *N. Engl. J. Med.*, **309**, 883.

BERTELSEN, K., HANSEN, M.K., PEDERSEN, P.H. & 4 others (1988). The prognostic and therapeutic value of second-look laparotomy in advanced ovarian cancer. *Br. J. Obstet. Gynaecol.*, **95**, 1231.

FASQUELLE, D., VILLEMAIN, D., MEFFRE, G. & AGNIUS-DELORD, C. (1988). Dosage du marqueur tumoral CA 125 dans les cancers de l'ovaire: comparaison des méthodes immunoenzymatique et immunoradiométrique. *Pathol. Biol.*, **36**, 225.

FINKLER, N.J., BENACERRAF, B., LAVIN, P.T., WOJCIECHOWSKI, C. & KNAPP, R.C. (1988). Comparison of serum CA 125, clinical impression, and ultrasound in the preoperative evaluation of ovarian masses. *Obstet. Gynecol.*, **72**, 659.

HAGLUND, C., HUHTALA, M.-L., HALILA, H. & 4 others (1986). Tumour-associated trypsin inhibitor, TATI, in patients with pancreatic cancer, pancreatitis and benign biliary diseases. *Br. J. Cancer*, **54**, 297.

HALILA, H., HUHTALA, M.-L., HAGLUND, C., NORDLING, S. & STENMAN, U.-H. (1987). Tumour-associated trypsin inhibitor (TATI) in human ovarian cyst fluid. Comparison with CA 125 and CEA. *Br. J. Cancer*, **56**, 153.

HALILA, H., LEHTOVIRTA, P. & STENMAN, U.-H. (1988). Tumour-associated trypsin inhibitor (TATI) in ovarian cancer. *Br. J. Cancer*, **57**, 304.

HUHTALA, M.-L., KAHANPÄÄ, K., SEPPÄLÄ, M., HALILA, H. & STENMAN, U.-H. (1983). Excretion of a tumor-associated trypsin inhibitor (TATI) in urine of patients with gynecological malignancy. *Int. J. Cancer*, **31**, 711.

KOELMA, I.A., NAP, M., RODENBURG, C.J. & FLEUREN, G.J. (1987). The value of tumour marker CA 125 in surgical pathology. *Histopathology*, **11**, 287.

MACDONALD, F., BIRD, R., STOKES, H., RUSSELL, B. & CROCKER, J. (1988). Expression of CEA, CA 125, CA 19-9 and human milk fat globule membrane antigen in ovarian tumours. *J. Clin. Pathol.*, **41**, 260.

MAUGHAN, T.S., FISH, R.G., SHELLEY, M., JASANI, B., WILLIAMS, G.T. & ADAMS, M. (1988). Antigen CA 125 in tumor tissue and serum from patients with adenocarcinoma of the ovary. *Gynecol. Oncol.*, **30**, 342.

MOGENSEN, O., MOGENSEN, B., JAKOBSEN, A. & SELL, A. (1988). Measurement of the ovarian cancer-associated antigen CA 125 prior to second look operation. *Eur. J. Cancer Clin. Oncol.*, **24**, 1835.

MOGENSEN, O., MOGENSEN, B. & JAKOBSEN, A. (1989*a*). Predictive value of CA 125 during early chemotherapy of advanced ovarian cancer. *Gynecol. Oncol.* (in the press).

MOGENSEN, O., MOGENSEN, B. & JAKOBSEN, A. (1989*b*). CA 125 in the diagnosis of pelvic masses. *Eur. J. Cancer Clin. Oncol.*, **25**, 1187.

NEUNTEUFEL, W. & BREITENECKER, G. (1988). Immunohistochemische Darstellung von CA 125, CA 19-9 und CEA in normalen und pathologisch veränderten Adnexen. *Geburtsh. Frauenheilk.*, **48**, 334.

NILOFF, J.M., KNAPP, R.C., LAVIN, P.T. & 6 others. (1986). The CA 125 assay as a predictor of clinical recurrence in epithelial ovarian cancer. *Am. J. Obstet. Gynecol.*, **155**, 56.

STENMAN, U.-H., HUHTALA, M.-L., KOISTINEN, R. & SEPPÄLÄ, M. (1982). Immunochemical demonstration of an ovarian cancer-associated urinary peptide. *Int. J. Cancer*, **30**, 53.

VAN DER BURG, M.E.L., LAMMES, F.B., VAN PUTTEN, W.L.J. & STOTER, G. (1988). Ovarian cancer: the prognostic value of the serum half-life of CA 125 during induction chemotherapy. *Gynecol. Oncol.*, **30**, 307.

VERGOTE, I.B., BØRMER, O.P. & ABELER, V.M. (1987). Evaluation of serum CA 125 levels in the monitoring of ovarian cancer. *Am. J. Obstet. Gynecol.*, **157**, 88.